# Wide variation in shape of hypoplastic left ventricles undergoing recruitment and biventricular repair: A statistical shape modeling study

**DOI:** 10.1016/j.jocmr.2024.101131

**Published:** 2024-12-06

**Authors:** Isabel R. Barnet, Noah E. Schulz, Sunil J. Ghelani, David M. Hoganson, Eric N. Feins, Peter E. Hammer, Sitaram M. Emani, Lynn A. Sleeper, Rebecca S. Beroukhim

**Affiliations:** a Harvard Medical School, Boston, Massachusetts, USA; b Department of Cardiovascular Surgery, Boston Children’s Hospital, Boston, Massachusetts, USA; c Department of Cardiology, Boston Children’s Hospital, Boston, Massachusetts, USA

**Keywords:** Hypoplastic left heart syndrome, Ventricular remodeling, Complex biventricular repair, Endocardial fibroelastosis, Statistical shape modeling, Principal component analysis

## Abstract

**Background:**

Patients with hypoplastic left ventricles (LV) who undergo volume-loading procedures (recruitment, biventricular [BIV] repair) are at risk for adverse outcomes, including heart failure and death. We investigated pre-BIV LV shape as a predictor of outcome after BIV repair in patients with hypoplastic LVs.

**Methods:**

Baseline and post-recruitment cardiac magnetic resonance imaging and computed tomography data were analyzed in patients with hypoplastic LV (<50 mL/m^2^). Statistical shape modeling (SSM) was utilized to generate a model of the shape and variability of LVs. Traditional measures of LV sphericity and eccentricity were also measured. Major adverse cardiovascular events (MACE) included heart failure, transplant, and death.

**Results:**

Of 95 patients with baseline mean LV volume 29 ± 13 mL/m^2^, 45/95 (47%) had a right dominant atrioventricular canal defect, 31/95 (33%) had a variant of hypoplastic left heart syndrome, and 18/95 (19%) had endocardial fibroelastosis (EFE). A wide variation in LV shape was found by SSM, and shape modes were associated with right ventricle (RV) and LV size, and diagnosis. BIV repair was achieved in 74/95 (78%) patients; 13/74 (18%) of BIV patients had MACE. Predictors of MACE following BIV repair included EFE, higher RV mass index, and higher RV end-diastolic volume index. No baseline or post-recruitment LV shape parameter was associated with the outcome after BIV repair.

**Conclusion:**

The shape model of hypoplastic LVs demonstrated a wide array of LV shapes. LVs gained sphericity and size and lost eccentricity with recruitment. Though the ventricles changed shape with recruitment, no specific LV shape characteristic at the baseline or post-recruitment stage was predictive of decision to proceed with BIV repair or outcome. Higher RV mass and volume may represent new biomarkers that predict outcomes following BIV repair in patients with hypoplastic LV. Further investigation could determine the reproducibility of these findings.

## Graphical abstract

**Figure:**
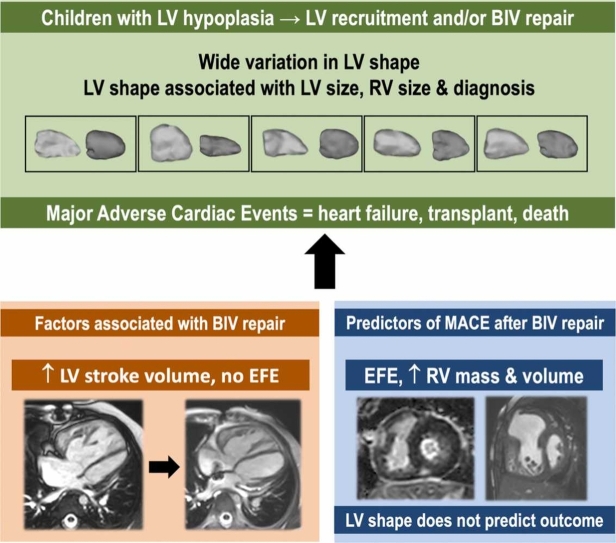

## 1 Introduction

Left ventricular (LV) hypoplasia is associated with a diverse group of congenital heart defects, such as variants of hypoplastic left heart syndrome (HLHS) with intact ventricular septum, unbalanced atrioventricular (AV) canal defects, and hypoplastic LV with ventricular septal defects and conotruncal abnormalities. Historically, many patients with these conditions have been surgically palliated with single ventricle strategies; however, the Fontan circulation is associated with short- and long-term adverse outcomes, including hepatic dysfunction, protein-losing enteropathy, and other organ dysfunction. As an alternative to Fontan, patients with hypoplastic LVs may undergo staged recruitment operations or primary biventricular (BIV) repair, both of which encourage growth of the LV [1]. Staged recruitment techniques, which involve increasing pulmonary blood flow and partially restricting the atrial septum to encourage flow through the LV, have been shown to increase LV volume, thereby allowing some patients to successfully undergo BIV repair [1], [2]. However, even with adequate volumetric growth of the LV with recruitment and subsequent BIV repair, a subset of patients may go on to develop heart failure characterized by systolic and/or diastolic dysfunction.

Previously defined risk factors for adverse outcomes after BIV repair have been identified as low LV end-diastolic volume index (EDVi), low left ventricle-to-right ventricle (LV:RV) stroke volume ratio, low LV stroke volume index (SVi), the presence of endocardial fibroelastosis (EFE), high preoperative LV end-diastolic pressure, and small mitral valve orifice area [3], [4], [5]. Although identification of these risk factors has improved patient selection for BIV repair, patients with hypoplastic LVs who meet existing criteria for BIV repair carry a 15% risk of ventricular dysfunction, heart failure, or death within a few years after BIV repair [6].

Prior studies of heart failure in adults have shown that LVs remodel by dilating and becoming more globular or spherical to maintain stroke volume; however, when the ejection fraction falls below 20%, further modulation of stroke volume is no longer possible through changes in ventricular architecture [7]. Similarly, children with dilated cardiomyopathy who develop progressive LV dilation and decreased wall thickness:end-diastolic dimension ratio are at greater risk for adverse outcomes [9]. However, little is known about whether hypoplastic LVs progress through adverse or healthy remodeling during ventricular recruitment. We hypothesized that a subgroup of patients with hypoplastic LVs who undergo recruitment and/or BIV repair may have LV shape characteristics that predispose them to postoperative heart failure, either at baseline or with volume loading. We also hypothesized that adverse remodeling following LV volume loading may parallel that seen in patients with dilated cardiomyopathy, with an increase in LV globularity or sphericity. Moreover, ventricular shape may be an important biomarker in congenital heart disease. Prior work has shown shape analysis of ventricles in congenital heart disease reveals important data about ventricular shape and remodeling not captured by conventional volumetric analysis of imaging data. For example, a comparison of patients with tricuspid atresia and Fontan against a population of normal controls identified abnormalities in LV shape, as well as correlations between a more spherical end-diastolic shape and decreased longitudinal shortening [10]. A preliminary analysis of patients with a variety of congenital heart diseases found that overall heart size and sphericalization of the heart were distinguishing shape differences compared to a population of normal controls, and important in predicting outcomes [11].

In this study, we performed an exploratory analysis of LV shape in patients with hypoplastic LVs undergoing LV recruitment and/or BIV repair. In addition to measurements of LV sphericity and eccentricity, we used three-dimensional (3D) imaging segmentation of the LVs to generate statistical shape models (SSMs) of the ventricles. SSM is a computational technique that converts geometric information of anatomy into a discrete representation that describes the object’s boundary. Using correspondence points on the surface of the shapes, the LVs can be co-registered/aligned and compared with each other. For a population of LVs, SSMs generate information about the average shape of the LVs and their geometric variation by finding numerous shape parameters (modes) that most efficiently describe the geometry of the ventricles in the population [12]. Such methods allow for the compression of complex geometric information into a smaller representation of shape and, therefore, augment traditional measurements of shape (eccentricity and sphericity) by providing more information. More information about SSM of the LV can be found through the Cardiac Atlas Project (Left Ventricular PCA Modes—Cardiac Atlas Project; www.cardiacatlas.org).

We sought to understand the variation in LV shape among our population of hypoplastic LVs and to determine if any LV shape characteristic might serve as a biomarker in predicting outcomes after BIV repair. Moreover, our primary aims were to (1) use SSM and traditional imaging-based measurements (eccentricity and sphericity) to characterize LV shape among patients with hypoplastic LVs; (2) quantify change in shape with LV recruitment; and (3) assess whether any traditional or SSM shape parameters are associated with outcome after BIV repair.

## 2 Methods

### 2.1 Subjects

A retrospective database search at Boston Children’s Hospital identified 95 patients at our institution with a hypoplastic LV (EDVi <50 mL/m^2^) between January 1, 2007 and December 31, 2023 who underwent either staged LV recruitment (e.g., atrial septal restriction with augmented pulmonary blood flow ± BIV completion resulting in LV growth) or single-stage BIV repair. The cohort included patients with unbalanced AV canal defects and variants of HLHS. All patients had a cardiovascular magnetic resonance (CMR) or cardiac computed tomography (CCT) imaging study at baseline, and a subset of patients had a follow-up study after staged LV recruitment. The highest resolution end-diastolic 3D CMR or CCT dataset available was used. If only systolic data were available, the study was excluded. Patients were also excluded if imaging did not include an end-diastolic full-volume dataset encompassing the entire LV. This retrospective study was approved by the Boston Children’s Hospital Institutional Review Board with waiver of consent (IRB-P00045572).

Most CMR images were acquired on a 1.5T scanner (Achieva or Ingenia, Philips Healthcare, Best, Netherlands) using general anesthesia, with the following parameters: either a balanced steady-state free precession axial cine stack at end-diastole or a 3D CMR image acquired at end-diastole was used for segmentation. Axial cine stacks were performed with breath-holding and electrocardiogram gating with typical slice thickness of 5–6 mm and typical spatial resolution of 1.7–2 mm × 1.7–2 mm. 3D balanced steady-state free precession whole-heart angiograms were typically used with either Gadavist (Bayer Healthcare, Leverkusen, Germany) or Ferumoxytol (Covis Pharma, Waltham, Massachusetts) contrast agents. Respiratory motion compensation was performed using a self-navigator to track and gate heart position. The typical reconstructed voxel size was on average 0.6 × 0.6 × 0.6 mm^3^ (near isotropic). Imaging parameters ranged between echo time 1.5–2.4 ms, repetition time 3.1–4.9 ms, flip angle 55–100°, and bandwidth 540–1575 Hz. Most CCT images were acquired using a third-generation dual-source computed tomography (CT) scanner at 70 kV (Somatom Force, Siemens Healthcare, Forchheim, Germany). For young patients unable to breath-hold who required cine imaging, retrospectively gated imaging was typically performed under general anesthesia. All other CCTs were performed as a prospectively triggered high-pitch spiral acquisition during diastole with a reconstructed spatial resolution of 0.6 mm slice thickness × 0.3 mm increment.

### 2.2 Clinical data

Clinical records were reviewed for demographic data, cardiac diagnosis, associated syndromes or other comorbidities, and outcomes, including circulation (e.g., BIV repair, 1.5V or reverse 1.5V repair, Fontan, transplant, Glenn with or without an aortopulmonary shunt, and stage 1 circulation) at last follow-up. A reverse 1.5V repair is a 1.5 ventricle-type circulation using the RV as the systemic ventricle and the LV as a pulmonary ventricle (carrying inferior vena cava flow) in addition to a Glenn shunt [13], [14] Diagnostic groups included (1) right dominant AV canal defect, (2) HLHS with intact ventricular septum and EFE, (3) HLHS with intact or nearly intact ventricular septum and no EFE, and (4) hypoplastic LV with moderate or large ventricular septal defect. EFE was also separately analyzed as a binary variable. Additional anatomic variables were collected, including the presence of a conotruncal abnormality (double outlet RV or transposition of the great arteries). Comorbidities that might increase the risk for adverse outcomes after Fontan operation were defined as heterotaxy syndrome, Down syndrome, pulmonary vein stenosis, and primary ciliary dyskinesia. Major adverse cardiovascular events (MACE) were defined as heart failure (heart failure service consultation or heart failure admission), heart transplantation, and death. Residual LV volume-loading lesions following BIV repair were defined as greater than or equal to moderate ventricular septal defect, greater than or equal to moderate mitral regurgitation, or greater than or equal to moderate aortic regurgitation.

### 2.3 CMR and CCT data

The following data were extracted from CMR and CCT images: right and left ventricular end-diastolic volume, end-systolic volume, mass, stroke volume, and ejection fraction. Ventricular mass and volumes were measured from CMR and CCT examinations by Simpson’s method of disks from ventricular short-axis planes. With the exception of the septal band, RV trabeculations were not included in RV mass calculations.

Two other measurements of LV shape were also collected: sphericity and eccentricity. LV end-diastolic length was measured to calculate the LV sphericity:

$$\mathrm{LV sphericity}=\frac{\mathrm{LVEDV}}{{\frac{4}{3}*\pi *(\frac{\mathrm{LVEDL}}{2})}^{3}}$$

where LVEDV = LV end-diastolic volume and LVEDL = LV end-diastolic length [15], [16] LV eccentricity was calculated as shown below, measured from an end-diastolic basal short-axis view:

$$\mathrm{LV eccentricity}=\frac{\mathrm{LV diameter parallel to septum}}{\mathrm{LV diameter perpendicular to septum}}$$

LV end-diastolic length was also indexed to the square root of body surface area for analysis as an independent variable.

### 2.4 Statistical shape modeling

**Fig. 1:**
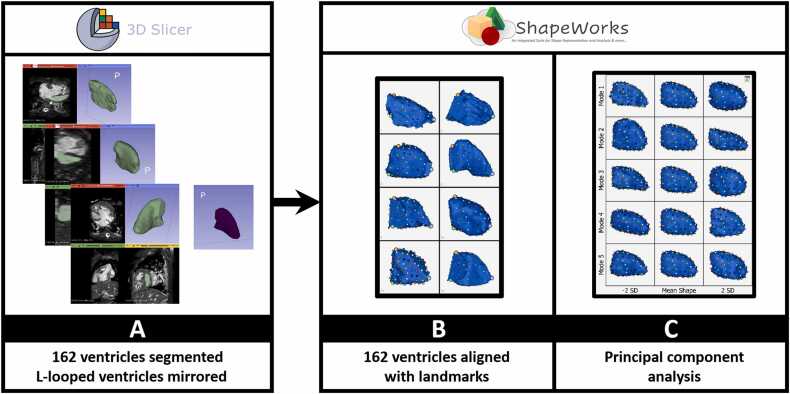
Image processing sequence for shape modeling principal component analysis. (A) DICOM source images at end-diastole were imported into 3D slicer for manual segmentation of the LV. L-looped ventricles were mirrored. (B) Segmentations were imported into ShapeWorks and aligned using landmarks, and a statistical shape modeling algorithm was applied to map correspondence points onto the LV surface. (C) Principal component analysis was applied to yield an average shape with individual shape parameters (modes 1-5 = M0-M4) that explain variance in shape across the group. *3D* three-dimensional, *DICOM* digital imaging and communications in medicine, *LV* left ventricle

SSM image processing steps are shown in Fig. 1. Three-dimensional models of the LVs at end-diastole were created from CMR and CCT images with 3D Slicer [17], an open-source image computing software developed at Brigham and Women’s Hospital. For both CMR and CCT images, 3D DICOM data were imported into 3D Slicer for segmentation. The boundary between the LV myocardium and blood pool was segmented manually, from apex to mitral annulus, as a single mask. For patients with AV canal defects, the boundary of the annulus was defined as the location where the plane of the ventricular septum crossed the plane of the common AV valve annulus. L-looped ventricles were mirrored so that all ventricles could be aligned and analyzed as a group. Because of a wide variation in conotruncal anatomy (e.g., in cases of double outlet RV, the only outflow was through a ventricular septal defect), the outflow tract was excluded from each segmentation.

To analyze LV shape, we utilized ShapeWorks [8], an open-source SSM software developed at the University of Utah. ShapeWorks uses a particle-based modeling (PBM) algorithm to generate a shape model of the population of ventricles and its variability. The PBM algorithm places particles (correspondence points) on the surface of each shape and optimizes their location to find the shape parameters, or modes, that most efficiently describe the geometry of the population. Each shape mode is an orthogonal direction of a principal components analysis (PCA) of the correspondence point positions. Moreover, each mode represents a particular shape characteristic and, thus, explains a certain percentage of the variation in shape across the population. Therefore, each PCA score can be analyzed as a metric that describes the magnitude of variation in ventricular shape relative to the population mean. PCA-based shape parameters allow for compression of a large amount of geometric information into a smaller representation of shape. There are typically a finite number of shape parameters that account for most of the variability in shape across the population.

We specifically applied PBM shape modeling to the cohort of all baseline and post-recruitment ventricles to generate modes that describe important shape characteristics. After importing the segmented ventricles from 3D Slicer to ShapeWorks, we spatially aligned the ventricles using anatomic landmarks (anterior mitral valve annulus, inferior mitral valve annulus, and apex) to ensure they were in the same orientation before shape analysis. Segmentations and spatial alignments were reviewed as a group to minimize error and maximize consistency. For the optimization phase to generate the shape modes, we used 128 surface correspondence points for each LV surface. Following completion of shape analysis, we extracted PCA scores of each ventricle for the first five shape modes (M0-4) for further statistical analysis. Change in shape with recruitment was computed as the difference in the mode score for a ventricle before and after recruitment for each mode. To identify broad shape characteristics of all LVs while also maintaining consistency and simplicity in SSM data throughout the manuscript, our primary SSM analysis included all baseline and post-recruitment ventricles. The entire SSM analysis was also repeated on just baseline ventricles (n = 95) to confirm that shape modes were similar to those of the primary SSM (n = 162) (Supplementary Material). Establishing this similarity validates the primary SSM modes as reliable descriptors of shape across groups.

## 3 Statistical analysis

Non-normally distributed continuous variables are described as median (interquartile range [IQR]), and normally distributed continuous variables are described as mean (standard deviation [SD]). Categorical variables are described as frequency (percentage). For continuous, normally distributed variables, a paired Student’s t-test was performed to compare parameters of ventricular size, function, LV sphericity, and LV shape among patients who underwent LV recruitment. Spearman’s correlation was used to compare LV sphericity, eccentricity, and other parameters of ventricular size and function with shape mode PCA scores (M0-4). Kruskal-Wallis test was used to compare baseline anatomic characteristics with baseline PCA mode scores. Univariate and multivariable logistic regression were performed to identify baseline factors associated with the decision to proceed toward BIV repair. Univariate and multivariable Cox proportional hazards regressions were used to identify factors associated with MACE events after BIV repair. Predictors significant at p < 0.15 in univariate analysis were included as candidate predictors in the multivariable modeling. Multivariable models were developed using backward stepwise regression. Predictors with p < 0.05 were retained in the final multivariable model. All tests were performed with a two-sided type I error rate of 0.05. Data analyses were performed with SAS software (version 9.4, SAS Institute, Cary, North Carolina).

## 4 Results

A search of the Heart Center database and complex BIV repair program database identified 95 children with hypoplastic LV who had undergone initial single ventricle palliation, followed by LV volume loading with ventricular recruitment and/or BIV repair. Baseline patient characteristics are shown in Table 1. The median age at the baseline study was 1.9 years (IQR 0.6, 3.4 years). The most common underlying diagnosis was right dominant AVC defect in 45 (47%), followed by HLHS variants in 31 (34%). Overall, 36 (38%) of patients had a comorbidity (e.g., heterotaxy syndrome, Down syndrome, pulmonary vein stenosis, primary ciliary dyskinesia) with increased risk of Fontan palliation. Of the entire group, 74 (78%) had an eventual BIV repair, (50 [53%] with LV recruitment and subsequent BIV repair + 24 (25%) with single-stage BIV repair). At last follow-up, none of the seven patients with a stage 1 or Glenn circulation are expected to undergo BIV repair. The median age at last follow-up was 6.7 years (IQR 4.6, 9.7 years). In follow-up, 18/95 (19%) of all patients and 13/74 (18%) of BIV patients had MACE (all patients: 11/95 [12%] with heart failure, 3/95 [3%] with transplant, and 4/95 [4%] with death; BIV patients: 8/74 [11%] with heart failure, 2/74 [3%] with transplant, and 3/74 [4%] with death). The median time to MACE after BIV repair was 3.3 years (IQR 1.1–6 years).

**Table 1:** Baseline characteristics (N = 95).

Variable	Measurement
Age at baseline, y	1.5 (0.6, 3.3)
Age at recruitment, y (N = 71)	2.2 (0.6, 3.4)
Age at BIV, y (N = 82)	3.1 (1.4, 4.7)
Age at last follow-up, y	6.7 (4.6, 9.7)
Male sex	56 (59)
L-looped ventricle	11 (12)
Diagnosis	
HLHS variant	31 (34)
HLHS with EFE	18 (19)
Hypoplastic LV with intact/near intact septum and no EFE	13 (14)
Hypoplastic LV with moderate or large VSD	19 (20)
Right dominant AV canal	45 (47)
Partial/transitional AV canal	11 (12)
Complete AV canal	34 (36)
Conotruncal abnormality (DORV/TGA)	33 (35)
Comorbidity	36 (38)
Heterotaxy syndrome	20 (21)
Down syndrome	6 (6)
Pulmonary vein stenosis	9 (9)
Primary ciliary dyskinesia	1 (1)
Other*	6 (6)
Recruitment operation	71 (74)
BIV repair	74 (78)
Single-stage BIV repair	24 (25)
Pacemaker	11 (12)
Circulation at last follow-up	
BIV	72 (76)
Reverse 1.5V	7 (7)
Fontan	5 (5)
Transplant	3 (3)
Glenn	2 (2)
Glenn with shunt	4 (4)
Stage 1	1 (1)
1.5V	1 (1)
Status at last follow-up	
Alive without heart failure or transplant	77 (81)
Alive with transplant	3 (3)
Alive with heart failure	11 (12)
Deceased	4 (4)

*HLHS* hypoplastic left heart syndrome, *LV* left ventricle, *EFE* endocardial fibroelastosis, *VSD* ventricular septal defect, *AV* atrioventricular, *DORV* double outlet right ventricle, *TGA* transposition of the great arteries, *BIV* biventricular, *1.5V* 1.5 ventricle, *IQR* interquartile range, *SD* standard deviation

Data expressed as N (%), median (IQR), or mean (SD)

* Other = hydronephrosis/eosinophilic esophagitis (n = 1); cleft lip/15q22.2 duplication of unclear significance (n = 1); esophageal atresia/tracheo-esophageal fistula (n = 1); VACTERL (N = 1); Pentalogy of Cantrell (n = 1); CHARGE syndrome (n = 1)

### 4.1 Conventional CMR and CCT parameters

For the baseline studies (N = 95), LV EDVi was 29 ± 13 mL/m^2^, with LV EF 60 ± 11%, LV end-diastolic length index 5.4 ± 0.9, LV sphericity of 0.53 ± 0.22, and LV eccentricity of 1.8 ± 0.5. RV EDVi was 105 ± 34 mL/m^2^, with RV mass index 35 ± 11 g/m^2^ and RV EF 55 ± 9%. The LV:RV EDV ratio was 0.3 ± 0.2 (Table 2). The median time from LV recruitment to post-recruitment imaging was 1 year (IQR 0.6–1.3 years). Significant changes in LV size, sphericity, and eccentricity were found with LV recruitment (N = 67), including increases in LV EDVi (+83%), SVi (+71%), LV:RV EDV ratio (+100%), sphericity (+17%), and length (+11%), with a decrease in LV eccentricity (−17%) (all p < 0.001; Table 2). Aside from a decrease in RV SVi, no other significant changes in RV mass and volume were observed following LV recruitment.

**Table 2:** Ventricular measurements.

Variable	All baseline N = 95	Pre-recruitment N = 67	Post-recruitment N = 67	p value*
Age, y	1.9 (0.6, 3.4)	2.3 (0.6, 3.1)	3.3 (2, 4.5)	<0.0001
LV mass index (g/m^2^)	25±9	25±10	35±14	<0.0001
LV EDVi (mL/m^2^)	29±13	28±13	53±27	<0.0001
LV mass:volume ratio	1.0±0.6	1.1±0.7	0.7±0.2	<0.0001
LV SVi (mL/m^2^)	17±9	17±9	29±13	<0.0001
LV EF (%)	60±11	59±12	57±14	0.169
RV mass index (g/m^2^)	35±11	35±11	33±12	0.206
RV EDVi (mL/m^2^)	105±34	103±30	107±46	0.447
RV SVi (mL/m^2^)	58±20	57±17	50±18	0.004
RV EF (%)	55±9	55±9	48±9	<0.0001
LV:RV EDV ratio	0.3±0.2	0.3±0.2	0.6±0.2	<0.0001
LV sphericity	0.53±0.22	0.51±0.22	0.62±0.22	<0.0001
LV eccentricity	1.8±0.5	1.8±0.5	1.5±0.3	<0.0001
LV length index (cm/m)	5.4±0.9	5.3±0.9	6.0±0.9	<0.0001
M0	−5±21	−5±21	7±18	<0.0001
M1	3±16	4±17	−4±18	<0.0001
M2	−0.4±12	−2±11	0.6±13	0.022
M3	2±10	1±10	−2±10	0.005
M4	−0.3±8	−0.3±8	0.4±9	0.252

*CMR cardiovascular magnetic resonance, CT computed tomography, LV left ventricle, EDVi end-diastolic volume index, SVi stroke volume index, EF ejection fraction, EDV end-diastolic volume, BIV biventricular, RV right ventricle, IQR interquartile range, SD standard deviation*

Data expressed as median (IQR) or mean ± SD

* Paired Student’s t-test for baseline and post-recruitment ventricles (N = 67). Baseline studies included 91 CMR + 4 CT. Pre-recruitment studies are a subset of baseline studies. Post-recruitment studies included 60 CMR + 7 CT

### 4.2 Statistical shape modeling

**Fig. 2:**
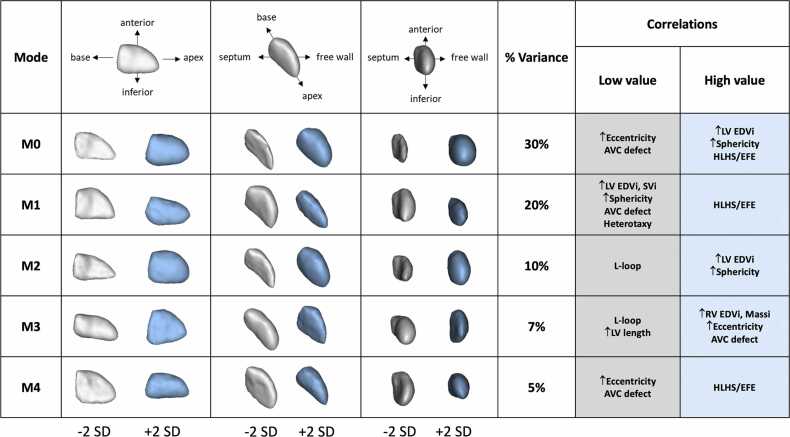
Results of the principal component analysis for all 162 LV shapes, % variance described by each component, and their associations. Each mode (M0-M4) represents a mathematically derived shape characteristic. The average LV shape is shown in the top row, with subsequent rows showing 2 standard deviations above and below the mean score for modes M0-M4. Additional modes (M5-M94) are not shown. *AVC* atrioventricular canal, *LV* left ventricle, *RV* right ventricle, *EDVi* end-diastolic volume index, *SVi* stroke volume index, *Massi* mass index, *HLHS/EFE* hypoplastic left heart syndrome with endocardial fibroelastosis

The shape modes generated by the SSM analysis of all baseline and post-recruitment ventricles (N = 162) are shown (Fig. 2, Video 1). Representative ventricular shapes for PCA scores at +2 and −2 SD from the mean PCA score for each mode are shown, with percent variance of total shape explained by each mode. The first five shape modes (M0-M4) explained 72% of the total variance in shape. The other shape modes (baseline M5-M161) are not shown because any mode after M4 explained <4% of variance. The aspect of shape described by each mode, outlined in Fig. 2, was defined by visual inspection and by correlation of mode scores with existing measures, such as volume and eccentricity. For baseline mode M0 (30% variance), the ventricle −2 SD from the mean shape is narrow/compressed, whereas the ventricle +2 SD from the mean is larger and more normal in shape (bullet-shaped). M0 was positively correlated with measured sphericity and negatively correlated with eccentricity (Supplemental Fig. 1). Other distinct variations in shape are captured by other modes, including changes in length, height, septal curvature, and apical shape (Fig. 2). Many LV shape modes were also correlated with other parameters, including underlying anatomical diagnosis, RV and LV size, eccentricity, and sphericity (Fig. 2, Video 1). Several of the associations were present across multiple shape modes. For example, LV EDVi correlated with a high score for M0, low score for M1, and high score for M2. The average scores for each mode in the baseline, pre- and post-recruitment ventricles are also shown (Table 2). Post-recruitment LVs had higher scores for M0 (Table 2, p < 0.001) and M2 (Table 2, p = 0.022) and lower scores for M1 (Table 2, p < 0.001) and M3 (Table 2, p < 0.005). These score differences represent increased sphericity, LV EDVi, and length following recruitment, similar to the changes measured in traditional imaging-based shape parameters (Table 2). A visual depiction of the change in LV shape with recruitment is shown in Video 2. Lastly, we also conducted individual shape analyses of the 95 baseline ventricles to ensure the modes characterizing this sub-population were comparable to those describing the combined population (Supplemental Fig. 2). The modes were similar between the two shape analyses.

### 4.3 Factors associated with BIV repair

Factors associated with decision to proceed with BIV repair are shown in Table 3. By univariate logistic regression, BIV repair was inversely associated with diagnosis of HLHS, the presence of EFE, and the need for a post-recruitment atrial septal stent; and BIV repair was positively associated with a higher (pre-recruitment) LV EDVi, LV SVi, and LV:RV EDV ratio, and a higher post-recruitment LV SVi. Absence of EFE and higher LV SVi were independent predictors of decision to proceed with BIV repair (adjusted odds ratios using baseline predictors: HLHS with EFE 0.1, [95% CI 0.02], 0.4; LV SVi 9.7 [95% CI 2.6, 36.5]; both p<0.001; C-statistic 0.89). Aside from a higher M2 score in pre-recruitment ventricles (representing D-looped ventricles with greater LV EDVi and sphericity), no shape characteristic of the baseline or post-recruitment ventricles was associated with eventual BIV repair.

**Table 3:** Factors associated with decision to proceed with BIV repair (N = 95).

Variable	BIV	No BIV	OR	95% CI	p value
Baseline variables	N = 74	N = 21			
Diagnosis					**0.002**
Right dominant AV canal	**38 (51%)**	**7 (33%)**	**Ref**		
HLHS with EFE	**7 (9%)**	**11 (52%)**	**0.1**	**0.04, 0.4**	
HLHS with no EFE	**12 (16%)**	**1 (5%)**	**1.6**	**0.2, 11.2**	
HLV with VSD	**17 (23%)**	**2 (10%)**	**1.4**	**0.3, 6.6**	
HLHS with EFE	**7 (9%)**	**11 (52%)**	**0.1**	**0.03, 0.3**	**<0.001**
LV massi*	25±7	28±12	0.7	0.4, 1.2	0.183
LV EDVi* (mL/m^2^)	**33±12**	**20±10**	**3.2**	**1.7, 5.9**	**<0.001**
LV mass:volume ratio†	**0.8±0.3**	**1.6±1**	**0.8**	**0.7, 0.9**	**0.001**
LV SVi† (mL/m^2^)	**20±8.2**	**11±5.3**	**8.8**	**2.9, 27**	**<0.001**
LV:RV EDV ratio†	**0.4±0.2**	**0.23±0.15**	**1.7**	**1.1, 2.7**	**0.009**
RV Massi* (g/m^2^)	35.3±11.8	35.3±8.7	1.0	0.6, 1.5	0.956
RV EDVi* (mL/m^2^)	104±35	101±28	1.0	0.9, 1.2	0.720
RV SVi* (mL/m^2^)	56.6±19.5	59.6±17.2	0.9	0.7, 1.2	0.507
LV shape					
LV length (cm/m)†	**5.6±0.8**	**4.9±0.8**	**2.9**	**1.4, 6.0**	**0.004**
LV sphericity†	0.6±0.3	0.5±0.2	1.1	0.9, 1.3	0.528
LV eccentricity†	1.8±0.4	1.8±0.8	0.9	0.4, 2.4	0.890
M0*	−6±19	−2±29	0.9	0.6, 1.1	0.380
M1*	1±15	8±20	0.8	0.6, 1.0	0.100
M2*	**1±11**	**−7±12**	**1.9**	**1.2, 3.1**	**0.009**
M3*	3±10	3±10	0.9	0.5, 1.4	0.570
M4*	1±9	1±9	0.7	0.4, 1.3	0.314
					
Post-recruitment variables	N = 46	N = 21			
ASD stent	**8 (17%)**	**11 (52%)**	**0.2**	**0.05, 0.6**	**0.003**
LV EDVi* (mL/m^2^)	56±21.2	50±37.6	1.1	0.9, 1.3	0.474
LV SVi* (mL/m^2^)	**32±11.8**	**23±13.0**	**2.1**	**1.2, 3.5**	**0.009**
RV Massi (g/m^2^)	33.2±10.9	35.1±14.8	0.9	0.6, 1.3	0.498
RV EDVi* (mL/m^2^)	106±42	115±52	1.0	0.9, 1.1	0.400
LV shape					
LV length (cm/m)†	6.2±0.9	5.7±1.0	1.9	0.8, 4.3	0.147
LV sphericity†	0.62±0.22	0.62±0.21	0.8	0.1, 8.8	0.850
LV eccentricity†	1.5±0.3	1.5±0.5	0.8	0.2, 3.7	0.748
M0*	7±17	8±21	1.0	0.7, 1.3	0.755
M1*	−5±18	−2±20	0.9	0.7, 1.2	0.542
M2*	−0.5±13	3±14	0.8	0.6, 1.2	0.337
M3*	−2±10	−2±10	1.0	0.6, 1.7	0.970
M4*	0.03±9	1±10	0.9	0.5, 1.5	0.643

Data expressed as numbers (%) or means ± standard deviation*, HLHS* hypoplastic left heart syndrome, *AV* atrioventricular, *DORV* double outlet right ventricle, *TGA* transposition of the great arteries, *VSD* ventricular septal defect, *EFE* endocardial fibroelastosis, *LV* left ventricle, *RV* right ventricle, *EDVi* end-diastolic volume index, *SVi* stroke volume index, *EF* ejection fraction, *ASD* atrial septal defect, *BIV* biventricular, *OR* odds ratio, *CI* confidence interval

M0-M4 principle component analysis modes.

Bolded variables have p value < 0.05

* Units = 10

† Units = 0.1

### 4.4 MACE following BIV repair

**Fig. 3:**
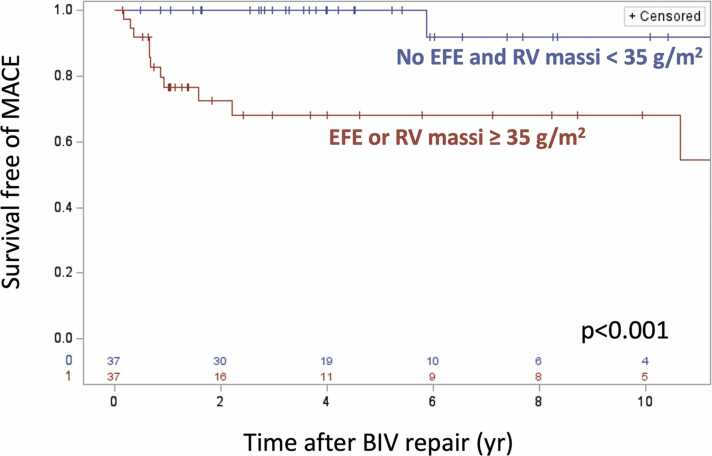
Kaplan-Meier survival curve for 74 patients who underwent BIV repair, stratified by the presence of EFE or RV mass index ≥35 g/m^2^. *EFE* endocardial fibroelastosis, *RV* right ventricle, *MACE* major adverse cardiac event, *BIV* biventricular

By univariate Cox regression, predictors of MACE following BIV repair (N = 74) included the presence of EFE, a higher baseline LV mass index, higher baseline RV mass index, RV EDVi, and RV SVi, and the presence of a post-BIV repair volume-loading lesion (Table 4). Using baseline variables, independent predictors of MACE included presence of EFE and higher baseline RV mass index (adjusted hazard ratios using baseline predictors: HLHS with EFE 3.9 [95% CI 1.2, 12.7], p=0.025; RV mass index 2.0 [95% CI 1.3, 3.2], p=0.002; Fig. 3). No shape characteristic, including traditional measures (eccentricity, sphericity) and SSM shape mode scores, of the baseline or post-recruitment ventricles predicted outcome after BIV repair (Table 4). Change in shape with recruitment did not predict BIV outcome (Table 4). A summary of the main study findings is provided in the Graphical Abstract.

**Table 4:** Univariate Cox regression for MACE for all BIV patients (N = 74).

Variable	MACE	No MACE	HR	95% CI	p value
Baseline characteristics	N = 13	N = 61			
Male sex	10 (77%)	31 (51%)	0.4	0.1, 1.5	0.172
L-loop ventricles	52 (85%)	11 (85%)	1.3	0.3, 6.3	0.702
Heterotaxy syndrome	2 (15%)	15 (25%)	0.7	0.1, 3.1	0.594
Partial AV canal	12 (92%)	52 (85%)	0.5	0.1, 3.7	0.488
Complete AV canal	10 (77%)	36 (59%)	0.4	0.1, 1.4	0.149
DORV/TGA	10 (77%)	34 (56%)	0.5	0.1, 2.0	0.332
VSD	**5 (38%)**	**47 (84%)**	**0.6**	**0.4, 1.0**	**0.031**
HLHS with EFE	**5 (38%)**	**2 (3%)**	**7.1**	**2.2, 22.4**	**<0.001**
Diagnosis					**0.011**
RDAVC	**4 (31%)**	**34 (56%)**	**Ref**		
HLHS with EFE	**5 (38%)**	**2 (3%)**	**9.2**	**2.3, 36.4**	
HLHS with no EFE	**2 (15%)**	**10 (16%)**	**2.1**	**0.4, 11.3**	
HLV with VSD	**2 (15%)**	**15 (25%)**	**1.6**	**0.3, 9.3**	
Comorbidity	4 (31%)	24 (39%)	0.8	0.2, 3.5	0.605
Single-stage BIV	5 (38%)	19 (31%)	0.9	0.3, 2.8	0.819
LV Massi (g/m^2^)*	**29.4±9.0**	**23.7±6.3**	**2.5**	**1.3, 5.0**	**0.009**
LV EDVi (mL/m^2^)*	34.1±7.2	33.0±12.8	1.1	0.7, 1.8	0.574
LV mass:volume ratio†	0.9±0.2	0.8±0.3	1.0	0.9, 1.2	0.692
LV SVi (mL/m^2^)*	20.8±5.0	19.9±8.8	1.2	0.6, 2.3	0.593
LV EF (%)†	61±8	60±8	1.2	0.6, 2.5	0.633
LV:RV EDV ratio†	0.29±0.09	0.4±0.2	0.8	0.5, 1.2	0.208
RV Massi (g/m^2^)*	**44.7±14.8**	**31.2±8.0**	**2.0**	**1.3, 3.0**	**0.001**
RV EDVi (mL/m^2^)*	**122±31**	**101±43**	**1.1**	**1.0, 1.3**	**0.029**
RV SVi (mL/m^2^)*	65.5±11.0	47.0±18.4	1.2	1.0, 1.5	0.056
RV EF (%)	54±6	48±8	0.9	0.4, 1.7	0.687
LV shape					
LV length (cm/m)†	5.7±1.0	5.6±0.8	1.6	0.7, 3.3	0.239
LV sphericity†	0.63±0.44	0.55±0.22	3.3	0.4, 27.1	0.273
LV eccentricity†	1.74±0.42	1.82±0.40	1.0	0.9, 1.1	0.532
M0*	−7±22	−6±18	1.0	0.8, 1.4	0.866
M1*	1±12	1±15	1.0	0.7, 1.5	0.943
M2*	6±14	0.4±10	1.4	0.8, 2.3	0.218
M3*	−1±10	2±10	0.8	0.4, 1.4	0.408
M4*	2±9	−1±8	1.4	0.7, 2.8	0.301
					
Post-recruitment shape	N = 6	N = 40			
∆ LV length†	0.8±0.7	0.8±0.6	1.0	0.9, 1.1	0.846
∆ LV sphericity†	0.07±0.11	0.11±0.18	0.9	0.6, 1.3	0.491
∆ LV eccentricity†	−0.17±0.22	−0.40±0.36	1.3	0.9, 1.8	0.213
∆ M0*	14±8	11±16	1.0	0.6, 1.8	0.996
∆ M1*	−14±15	−6±14	1.1	0.6, 2.0	0.826
∆ M2*	−2±9	1±9	1.7	0.8, 3.7	0.198
∆ M3*	2±8	−4±10	0.7	0.4, 1.3	0.287
∆ M4*	−4±5	3±9	2.1	1.0, 4.5	0.590
					
Post-BIV volume lesion	**8/13 (62)**	**14/61 (23)**	**3.6**	**1.2, 11.5**	**0.026**

Data expressed as numbers (%) or means ± standard deviation*, HLHS* hypoplastic left heart syndrome, *AV* atrioventricular, *DORV* double outlet right ventricle, *TGA* transposition of the great arteries, *VSD* ventricular septal defect, *EFE* endocardial fibroelastosis, *HLV* hypoplastic left ventricle, *RDAVC* right dominant atrioventricular canal, *BIV* biventricular, *EDVi* end-diastolic volume index, *SVi* stroke volume index, *EF* ejection fraction, *ASD* atrial septal defect, *EDV* end-diastolic volume, *MACE* major adverse cardiac event, *RV* right ventricle.

Post-recruitment LV shape predictors are shown in Supplemental Table 1

Bolded variables have p value < 0.05

* Per 10 unit increase

† Per 0.1 unit increase

## 5 Discussion

The present study aimed to evaluate LV shape as a biomarker of outcome in a cohort of patients with hypoplastic LV who underwent subsequent volume loading through LV recruitment and/or BIV repair. We found a wide variation in LV shape among our population, including compressed ventricles, bullet-shaped ventricles, differences in the configuration and morphology of the ventricular apex, and varied indexed lengths and dimensions. Using PCA, we were able to quantify the variation in shape among this large cohort of patients to identify the most common differences. The first mode (M0), which represented the difference between a compressed small ventricle and a bullet-shaped larger ventricle, accounted for the most (30%) variance in ventricular shape. We found multiple associations between the shape modes (M0-M4) and underlying diagnosis, as well as other more traditional parameters of ventricular size and shape. Following recruitment, LVs remodeled with an increase in volume and sphericity and decrease in eccentricity. No shape parameter was independently associated with decision proceed toward BIV repair, nor predictive of outcome after BIV repair. Furthermore, no change in shape after LV recruitment predicted outcome after BIV repair. The RVs for this cohort were dilated and hypertrophied, with minimal changes in end-diastolic volume, mass, and stroke volume following recruitment. Although no RV parameter was associated with decision to proceed toward BIV repair, elevated RV mass and RV EDVi were associated with higher risk of MACE following BIV repair.

As demonstrated by our shape analysis, many of the LVs in our cohort that began as small, compressed ventricles had the capacity to undergo healthy remodeling and ultimately function well as a systemic LV. Thus, it appears that LV shape is neither a surrogate for myocardial health/remodeling potential nor an important factor when evaluating candidacy for BIV repair. There are multiple potential factors explaining these findings. First, our patients at baseline had single ventricle physiology, and thus the analyzed LVs were not exposed to a full cardiac output. With LV recruitment, ventricles dilate and increase stroke volume as predicted by the Frank-Starling mechanism [2]. However, volume loading in some cases will unmask non-compliant features of a ventricle that were previously unrecognized. Moreover, a high preoperative LV end-diastolic pressure has been shown to predict adverse outcomes after BIV repair, but the sensitivity of this marker is limited in the preoperative, LV volume-unloaded patient [5]. Although our study did not identify change in LV shape with volume loading as a predictor of outcome, our comparison cohort size may have been too small to achieve statistical significance, and the relative degree of volume loading likely varied among different patients. In addition, we will typically dilate the atrial septum in patients with high left atrial pressures; therefore the recruited ventricles in our cohort were likely protected against excessive volume loading. It is also possible that some of the LVs in our study progressed to adverse remodeling with more significant changes in LV shape following BIV repair. Although future studies could investigate post-BIV repair shape as a predictor of outcome, such investigations would not inform clinicians on decision-making regarding candidacy for BIV repair. Finally, many of our patients underwent complex BIV repair, involving multiple surgical interventions such as mitral valvuloplasty, arterial switch operation, and ventricular septal defect closure, with a 12% risk of pacemaker after LV recruitment or BIV repair. Our finding that the presence of a post-BIV volume-loading lesion conferred >3-fold risk in adverse outcomes after BIV repair suggests that residual lesions in the setting of vulnerable myocardium are important factors in the development of heart failure in this population.

Prior work using SSM in congenital and acquired heart disease has shown important correlations between cardiac shape and function. One such study demonstrated that several RV shape modes in patients with tetralogy of Fallot correlate with systolic hemodynamic forces in the RV and exercise capacity [18]. In addition, adults with dilated left atria, as assessed by SSM, carry a higher risk of atrial fibrillation recurrence after ablation, and a higher risk of left atrial thrombogenesis [19], [20]. Other non-SSM shape studies have shown that increased LV sphericity has prognostic value in patients with dilated cardiomyopathy [15], [21] Although the shape variations identified by these prior investigations appear to be markers of disease, they are most likely indicators of progression in adverse cardiac remodeling rather than upstream, causative factors in each disease process. The precise mechanisms and pathophysiology behind ventricular remodeling remain controversial, and likely relate to the complex interplay between chemical signaling pathways, neurohormonal activation, the extracellular matrix, oxidative stress, myocyte hypertrophy, and cell death, as well as mechanical factors including ventricular hypertrophy and wall stress. [22] Myocardial volume and function, which play an integral role in the pathophysiology of heart failure, are not captured by assessment of many of the static global shape parameters assessed by SSM. In our univariate analysis, the only LV parameter that predicted outcome after BIV repair was a higher LV mass index. Thus, more detailed SSM techniques that assess characteristics of the myocardium in addition to ventricular chamber shape throughout the cardiac cycle, have the potential to shed more light on patterns of healthy versus adverse remodeling in congenital heart disease.

Although our dataset describes the variation in LV shape among patients with hypoplastic LVs, it does not provide insight into how hypoplastic LVs differ from a population of normal LVs. To address this, investigators working with the Cardiac Atlas Project have developed a methodology for the development of z-scores for each shape mode, relative to a population of normal controls [10]. Further developments in this process, allowing for more standardization of shape modes and z-scores, could allow for increased clinical relevance of SSM in the assessment of patients with congenital heart disease.

Congruent with the theory that the LV size, shape, and function cannot be isolated from the RV, we found that RV mass index decreased with LV volume loading, and higher baseline RV mass index and RV EDVi were associated with MACE following BIV repair. The same held true for the post-recruitment predictors of MACE, which included a higher post-recruitment RV mass index and higher post-recruitment RV SVi. As the RV is the main ventricular pump in the baseline physiology of a patient with hypoplastic LV, a higher RV EDVi and higher RV mass index at baseline suggest that the RV may be sustaining a higher workload that will ultimately require transfer to the LV following BIV conversion. Thus, RV parameters of mass and volume may allow for prediction of the load required of the LV following BIV repair; and a larger, thicker RV may reflect a larger anticipated load. Our data suggest that clinicians could be more discriminatory in patients with elevated RV mass and EDVi before recommending LV recruitment and/or BIV repair, using the LV as a systemic ventricle. Therefore, future work might center on exploring RV mass, size, and shape as predictive parameters for hypoplastic LV patients undergoing LV recruitment and BIV repair. For patients who are deemed inappropriate candidates for standard BIV repair with a systemic LV, alternative circulations, such as reverse BIV circulation with a systemic RV and pulmonary LV, reverse 1.5 ventricle circulation with a systemic RV, Glenn, and pulmonary LV, or Fontan, could be considered.

## 6 Limitations

Given that this was a retrospective study, we were limited to patients who had baseline CMR or CT prior to LV recruitment or BIV repair. We also cannot measure the time frame in which LV size, shape, and function evolved. Our data support the notion that, in the overall cohort, clinical teams chose not to pursue BIV repair in patients with unfavorable LV characteristics, as patients with lower LV EDVi, LV SVi, LV:RV EDV ratio, and EFE were significantly less likely to undergo BIV repair. Thus, selection bias in the patients who ultimately underwent BIV repair may explain why few traditional LV parameters were associated with adverse outcome following BIV repair. We were also limited to available imaging sequences; therefore, the source data for shape models were varied in modality (CMR versus CCT), imaging sequence used for 3D models, and image resolution. For example, we were unable to control for the exact phase of the cardiac cycle for the single-phase 3D datasets; and therefore these images may not have been acquired precisely at end-diastole. We chose to measure LV shape at end-diastole because of the wider availability of end-diastolic full-volume images. However, it is possible that end-systolic shape may be clinically useful. Furthermore, assessment of RV shape could provide additional insight into the mechanisms of healthy versus adverse remodeling in our patient cohort. The small sample size also limits power to identify predictor variables and precise cutoffs for predictive modeling. Nonetheless, this study was novel in its application of robust SSM methods to a cohort of anatomically complex LVs and identification of new RV biomarkers for adverse outcomes.

## 7 Conclusions

A wide variation in shape of hypoplastic LVs was found among patients undergoing ventricular recruitment and/or BIV repair. Though the ventricles changed shape with recruitment, no specific LV shape characteristic was predictive of outcome at the baseline or post-recruitment stage. Patients with EFE, larger baseline RV end-diastolic volume, and larger RV mass index had higher likelihood of reaching an adverse outcome following BIV repair. Further work is needed to guide individualized decision-making about the most appropriate destination circulation for this complex and diverse group of patients.

## Funding

Not applicable.

## Author contributions

I.R.B.: Conception, design, data acquisition, analysis, interpretation, manuscript preparation and revision. N.E.S.: Conception, design, interpretation, manuscript revision. S.J.G.: Conception, design, manuscript revision. D.M.H.: Conception, design, manuscript revision. E.N.F.: Conception, design, manuscript revision. P.E.H.: Conception, design, manuscript revision. S.M.E.: Conception, design, manuscript revision. L.A.S.: Conception, design, manuscript revision. R.S.B.: Conception, design, data analysis, interpretation, manuscript preparation and revision. All authors have approved the submitted version and have agreed both to be personally accountable for the author’s own contributions and to ensure that questions related to the accuracy or integrity of any part of the work, even ones in which the author was not personally involved, are appropriately investigated, resolved, and the resolution documented in the literature.

## Ethics approval and consent

The Boston Children’s Hospital Institutional Review Board approved the study (IRB-P00045572) and informed consent was waived.

## Consent for publication

Not applicable.

## Declaration of competing interests

The authors declare that they have no known competing financial interests or personal relationships that could have appeared to influence the work reported in this paper.

## Data Availability

The datasets used and/or analyzed during the current study are available from the corresponding author upon reasonable request.

## Footnotes

Appendix A Supplementary data associated with this article can be found in the online version at doi:10.1016/j.jocmr.2024.101131.
