# Supplementary figures and images for: Wide variation in shape of hypoplastic left ventricles undergoing recruitment and biventricular repair: A statistical shape modeling study
Source: J Cardiovasc Magn Reson. 2024 Dec 6;27(1):101131. doi: 10.1016/j.jocmr.2024.101131 (PMC11780089; doi:10.1016/j.jocmr.2024.101131)

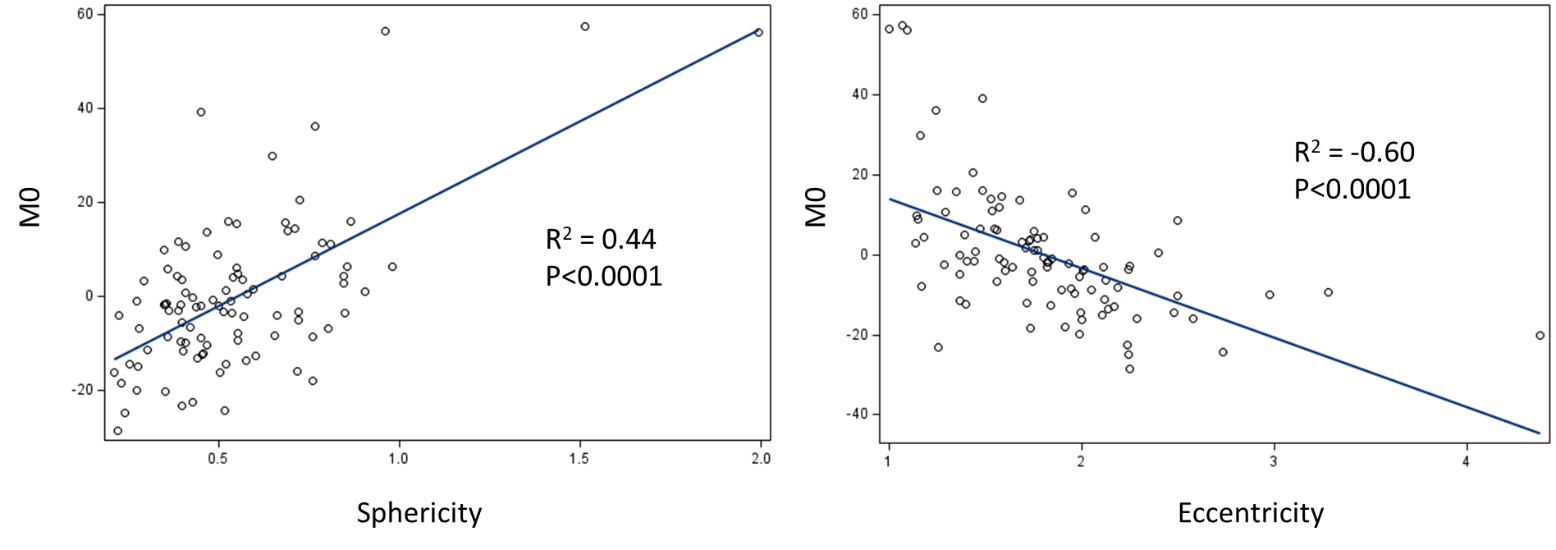


Supplemental Figure 1. Correlations between M0 PCA scores, LV sphericity and LV eccentricity.

Supplement: Supplementary file 1 — Supplementary material [file mmc1.docx]
