# Supplementary material for: Wide variation in shape of hypoplastic left ventricles undergoing recruitment and biventricular repair: A statistical shape modeling study
Source: J Cardiovasc Magn Reson. 2024 Dec 6;27(1):101131. doi: 10.1016/j.jocmr.2024.101131 (PMC11780089; doi:10.1016/j.jocmr.2024.101131)
Supplement: Supplementary file 2 — Supplementary material [file mmc2.docx]

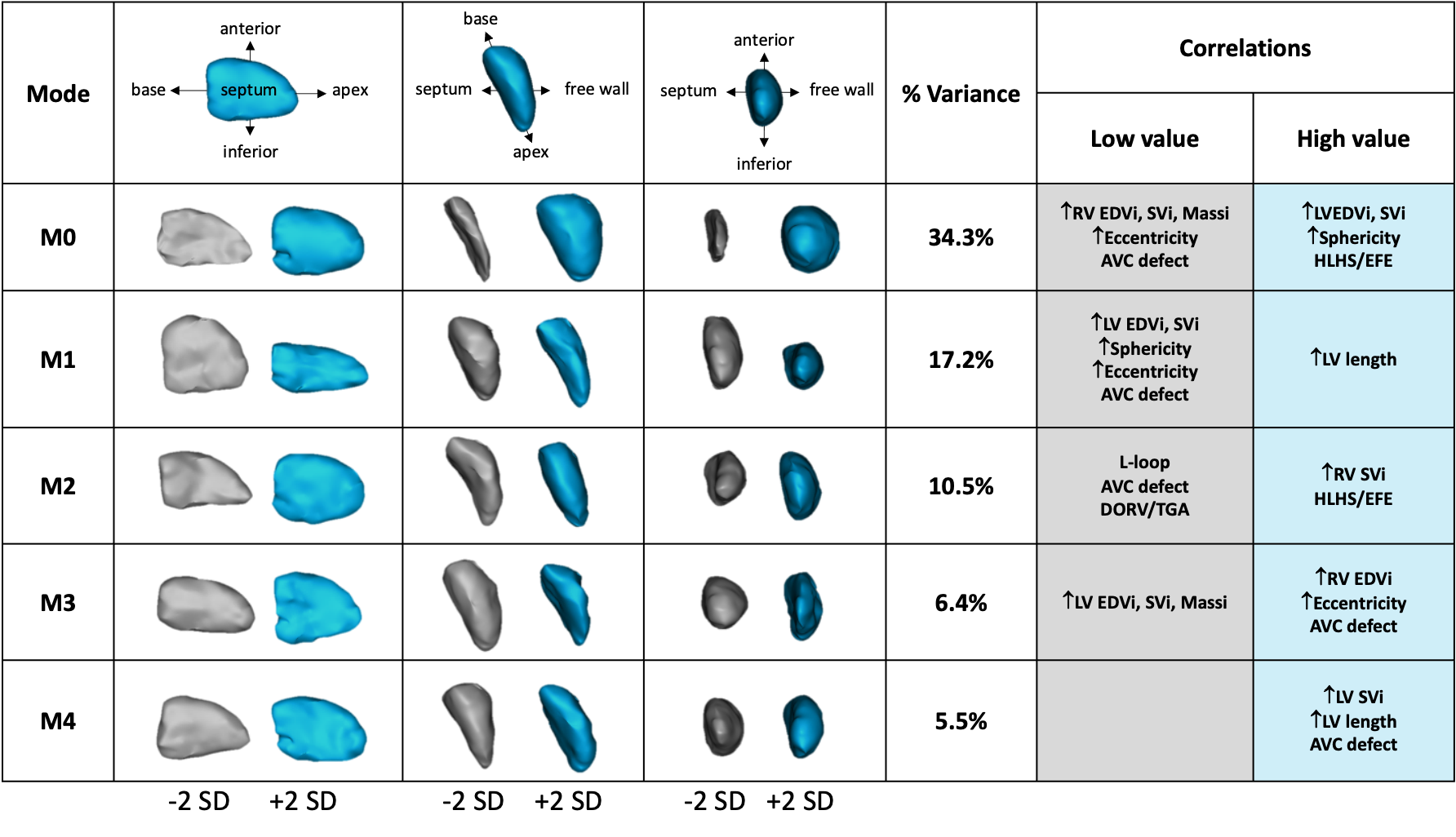


Figure 2. Results of the principal component analysis for the 95 baseline LV shapes, % variance described by each component, and their associations. Each mode (M0-M4) represents a mathematically derived shape characteristic. The average LV shape is shown in the top row, with subsequent rows showing 2 standard deviations above and below the mean score for modes M0-M4. Additional modes (M5-M94) are not shown. AVC = atrioventricular canal; LV = left ventricle; RV = right ventricle, EDVi = end-diastolic volume index; SVi = stroke volume index, Massi = mass index; HLHS/EFE = hypoplastic left heart syndrome with endocardial fibroelastosis; DORV/TGA = double outlet right ventricle or transposition of the great arteries.
