# Supplementary material for: Wide variation in shape of hypoplastic left ventricles undergoing recruitment and biventricular repair: A statistical shape modeling study
Source: J Cardiovasc Magn Reson. 2024 Dec 6;27(1):101131. doi: 10.1016/j.jocmr.2024.101131 (PMC11780089; doi:10.1016/j.jocmr.2024.101131)
Supplement: Supplementary file 3 — Supplementary material [file mmc3.docx]

Table 4. Univariate Cox regression for MACE for all BIV patients (N=74).

| Variable | MACE | No MACE | HR | 95% CI | P value |
| --- | --- | --- | --- | --- | --- |
| **Baseline characteristics** | **N=13** | **N=61** |  |  |  |
| Male sex | 10 (77%) | 31 (51%) | 0.46 | 0.12, 1.73 | 0.253 |
| L-loop ventricles | 52 (85%) | 11 (85%) | 1.5 | 0.3, 7.1 | 0.591 |
| Heterotaxy syndrome | 2 (15%) | 15 (25%) | 0.7 | 0.2, 3.2 | 0.654 |
| Partial AV Canal | 12 (92%) | 52 (85%) | 0.6 | 0.1, 4.4 | 0.583 |
| Complete AV Canal | 10 (77%) | 36 (59%) | 0.3 | 0.1, 1.3 | 0.113 |
| DORV/TGA | 10 (77%) | 34 (56%) | 0.5 | 0.1, 1.8 | 0.296 |
| **VSD** | **5 (38%)** | **47 (84%)** | **0.6** | **0.4, 0.9** | **0.017** |
| **HLHS with EFE** | **5 (38%)** | **2 (3%)** | **6.6** | **2.1, 20.8** | **0.001** |
| **Diagnosis** |  |  |  |  |  |
| **RDAVC** | **4 (31%)** | **34 (56%)** | **Ref** |  | **0.015** |
| **HLHS with EFE** | **5 (38%)** | **2 (3%)** | **9.2** | **2.2, 38.6** |  |
| **HLHS with no EFE** | **2 (15%)** | **10 (16%)** | **2.3** | **0.4, 14.1** |  |
| **HLV with VSD** | **2 (15%)** | **15 (25%)** | **0.8** | **0.1, 5.4** |  |
| Comorbidity | 4 (31%) | 24 (39%) | 0.5 | 0.2, 2.0 | 0.358 |
| Single stage BIV | 5 (38%) | 19 (31%) | 0.9 | 0.3, 3.1 | 0.908 |
| **LV Massi†** | **29.4 ± 9.0** | **23.7 ± 6.3** | **2.7** | **1.4, 5.3** | **0.004** |
| LV EDVi† | 34.1 ± 7.2 | 33.0 ± 12.8 | 1.2 | 0.7, 1.9 | 0.501 |
| LV mass:volume ratio‡ | 0.9 ± 0.2 | 0.8 ± 0.3 | 1.1 | 0.9, 1.2 | 0.484 |
| LV SVi† | 20.8 ± 5.0 | 19.9 ± 8.8 | 1.3 | 0.6, 2.4 | 0.504 |
| LV EF | 61 ± 8 | 60 ± 8 | 10.6 | NE, NE | 0.539 |
| LV:RV EDV ratio | 0.29 ± 0.09 | 0.4 ± 0.2 | 0.1 | NE, 3.6 | 0.178 |
| **RV Massi†** | **44.7 ± 14.8** | **31.2 ± 8.0** | **2.1** | **1.4, 3.2** | **<0.001** |
| **RV EDVi†** | **121.8 ± 31.3** | **100.5 ± 42.9** | **1.2** | **1.1, 1.3** | **0.008** |
| **RV SVi†** | **65.5 ± 11.0** | **47.0 ± 18.4** | **1.3** | **1.0, 1.6** | **0.022** |
| RV EF | 54 ± 6 | 48 ± 8 | 0.1 | NE, 90 | 0.466 |
| **Baseline LV shape** |  |  |  |  |  |
| LV sphericity‡ | 0.63 ± 0.44 | 0.55 ± 0.22 | 3.3 | 0.4, 27.1 | 0.273 |
| LV eccentricity‡ | 1.74 ± 0.42 | 1.82 ± 0.40 | 1.0 | 0.9, 1.1 | 0.532 |
| LV ED length index‡ | 5.7 ± 1.0 | 5.6 ± 0.8 | 1.6 | 0.7, 3.3 | 0.239 |
| M0† | 3.1 ± 19.6 | 0.5 ± 14.3 | 1.2 | 0.8, 1.6 | 0.375 |
| M1† | -0.4 ± 11.7 | -1.0 ± 11.6 | 1.2 | 0.7, 2.1 | 0.471 |
| M2† | 3.3 ± 7.8 | -1.4 ± 8.3 | 1.9 | 0.9, 3.9 | 0.080 |
| M3† | -0.9 ± 8.1 | 0.1 ± 6.7 | 0.9 | 0.4, 2.1 | 0.758 |
| M4† | 0.3 ± 5.3 | 1.0 ± 6.6 | 0.9 | 0.4, 2.4 | 0.880 |
| **Post recruitment LV shape** | **N=8** | **N=40** |  |  |  |
| LV sphericity‡ | 0.56 ± 0.18 | 0.62 ± 0.22 | 0.9 | 0.6, 1.3 | 0.519 |
| LV eccentricity‡ | 1.60 ± 0.4 | 1.46 ± 0.24 | 1.3 | 0.9, 1.9 | 0.146 |
| LV ED length index‡ | 6.5 ± 1.1 | 6.1 ± 0.8 | 1.0 | 0.9, 1.1 | 0.621 |
| M0’† | 1.6 ± 9.4 | 0.1 ± 21.7 | 0.8 | 0.3, 1.8 | 0.554 |
| M1’† | -1.6 ± 12.4 | -5.7 ± 16.9 | 1.3 | 0.7, 2.3 | 0.468 |
| M2’† | -0.6 ± 9.4 | 0.5 ± 15.0 | 1.2 | 0.5, 2.7 | 0.731 |
| M3’† | 2.1 ± 6.9 | 0.5 ± 7.2 | 1.5 | 0.5, 4.6 | 0.481 |
| M4’† | -2.4 ± 3.9 | -0.2 ± 10.2 | 0.9 | 0.3, 2.7 | 0.872 |
| **∆ LV shape with recruitment** |  |  |  |  |  |
| ∆ LV sphericity‡ | 0.07 ± 0.11 | 0.11 ± 0.18 | 0.9 | 0.6, 1.4 | 0.723 |
| ∆ LV eccentricity‡ | -0.17 ± 0.22 | -0.40 ± 0.36 | 1.3 | 0.9, 1.9 | 0.176 |
| ∆ M0’ | -3.7 ± 7 | -1.6 ± 23.5 | 1.0 | 0.6, 1.6 | 0.914 |
| ∆ M1’ | 9.8 ± 10 | 9.4 ± 12.4 | 1.2 | 0.5, 2.5 | 0.687 |
| ∆ M2’ | -0.5 ± 4.4 | -1.5 ± 13.6 | 1.3 | 0.5, 3.6 | 0.650 |
| ∆ M3’ | -0.8 ± 6.5 | -1.3 +- 8.3 | 1.6 | 0.3, 8.7 | 0.605 |
| ∆ M4’ | 0.9 ± 4 | 0.5 ± 10.9 | 0.7 | 0.1, 3.7 | 0.657 |
| **Post BIV volume lesion** | **8/13 (62)** | **14/61 (23)** | **3.7** | **1.2, 11.3** | **0.025** |

† per 10 unit increase, ‡ per 0.1 unit increase. HLHS = hypoplastic left heart syndrome; AV = atrioventricular; DORV = double outlet right ventricle; TGA = transposition of the great arteries; VSD = ventricular septal defect; EFE = endocardial fibroelastosis; HLV = hypoplstic left ventricle; RDAVC = right dominant atrioventricular canal; BIV = biventricular; EDVi = end-diastolic volume index; SVi = stroke volume index, EF = ejection fraction; ASD = atrial septal defect; EDV = end-diastolic volume; MACE = major adverse cardiac event.
